# Endurance Training Inhibits Insulin Clearance and IDE Expression in Swiss Mice

**DOI:** 10.1371/journal.pone.0118809

**Published:** 2015-03-30

**Authors:** José M. Costa-Júnior, Sandra M. Ferreira, André O. Protzek, Gustavo J. Santos, Ana P. Cappelli, Leonardo R. Silveira, Cláudio Zoppi, Camila A. M. de Oliveira, Antonio C. Boschero, Everardo M. Carneiro, Luiz F. Rezende

**Affiliations:** 1 Department of Structural and Functional Biology, Institute of Biology, State University of Campinas (UNICAMP), P.O. Box 6109, Campinas, SP, CEP 13083-865, Brazil; 2 Department of Biochemistry and Immunology, Faculty of Medicine of Ribeirão Preto, University of Sao Paulo (USP), Ribeirão Preto, SP, Brazil; 3 Department of Biosciences, Federal University of Sao Paulo (Unifesp), Santos, SP, CEP 11060-001, Brazil; CRCHUM-Montreal Diabetes Research Center, CANADA

## Abstract

**Introduction:**

Endurance training improves peripheral insulin sensitivity in the liver and the skeletal muscle, but the mechanism for this effect is poorly understood. Recently, it was proposed that insulin clearance plays a major role in both glucose homeostasis and insulin sensitivity. Therefore, our goal was to determine the mechanism by which endurance training improves insulin sensitivity and how it regulates insulin clearance in mice.

**Methods:**

Mice were treadmill-trained for 4 weeks at 70–80% of maximal oxygen consumption (VO_2_ max) for 60 min, 5 days a week. The glucose tolerance and the insulin resistance were determined using an IPGTT and an IPITT, respectively, and the insulin decay rate was calculated from the insulin clearance. Protein expression and phosphorylation in the liver and the skeletal muscle were ascertained by Western blot.

**Results:**

Trained mice exhibited an increased VO_2_ max, time to exhaustion, glucose tolerance and insulin sensitivity. They had smaller fat pads and lower plasma concentrations of insulin and glucose. Endurance training inhibited insulin clearance and reduced expression of IDE in the liver, while also inhibiting insulin secretion by pancreatic islets. There was increased phosphorylation of both the canonical (IR-AKT) and the non-canonical (CaMKII-AMPK-ACC) insulin pathways in the liver of trained mice, whereas only the CaMKII-AMPK pathway was increased in the skeletal muscle.

**Conclusion:**

Endurance training improved glucose homeostasis not only by increasing peripheral insulin sensitivity but also by decreasing insulin clearance and reducing IDE expression in the liver.

## Introduction

Insulin resistance is characterized by decreased insulin action on peripheral organs (primarily the liver and the skeletal muscle) and reduced phosphorylation and activation of the insulin receptor (IR), IRSs and components of the phosphatidylinositol 3-kinase/protein kinase B (AKT) pathway. Over time, beta cells lose their functionality, as evidenced by a reduction in glucose-stimulated insulin secretion (GSIS) and a loss of beta cell mass [1], which results in hyperglycemia.

Previous data indicate that the clearance of insulin predominantly depends on its degradation by the liver and that this clearance plays a critical role in glycemic control [2]. In hepatocytes, insulin is degraded by insulin-degrading enzyme (IDE) [3].

Exercise training protects against detrimental changes in glucose metabolism [4]. Its beneficial effects include increased insulin sensitivity in peripheral organs [5, 6], increased VO_2_ max [7–9], increased muscle microvascular perfusion [10] and lower body weight [11].

Nevertheless, there are no data on the effects of endurance training on insulin clearance despite the pivotal importance of this process in the regulation of glycaemia and of exercise in glucose regulation [12].

The aim of this study was to evaluate the effects of endurance training on insulin clearance and find possible mechanisms for these effects, such as IDE expression.

## Materials and Methods

### Reagents and solutions

Primary antibodies used for Western blotting: anti-phospho-AMPKα2^Thr172^, anti-AMPKα2, anti-phospho-acetyl-CoA carboxylase^Ser79^, and anti-ACC (Cell Signaling Technology, Boston, MA, USA); antiphospho-CaMKII^Tyr305^ and anti-CaMKII (Abcam); anti-IDE, anti-GAPDH, anti-phospho-IR, IR, anti-phospho-AKT, and AKT (Santa Cruz Biotechnology).

### Animals

8–12 weeks-old male Swiss mice (Unib:SW strain) acquired from the State University of Campinas were maintained on a 12 h light–dark cycle at 20–21°C with controlled humidity during the entire experiment and were fed a standard CHOW diet and offered tap water *ad libitum*. All experiments adhered to ACSM recommendations and were approved by State University of Campinas Ethics Committee.

#### Maximal oxygen consumption (VO_2_ max) and the endurance training protocol

Before measuring the VO_2_ max, mice underwent a one-week adaptation to the treadmill and running procedures. During this adaptation week, animals ran at 0.3 km.h^-1^ for 5 min each day. The VO_2_ max was measured in individual sealed treadmills at a 25° incline that were coupled to a gas analyzer (Oxylet system, Pan Lab/Harvad Instruments, Spain) immediately before and after the endurance training protocol was completed.

Mice warmed up for 8 min at 15 cm.sec^-1^. Subsequently, the treadmill speed was increased by 10 cm.sec^-1^ each minute until the mice were not able to maintain the necessary effort level. Oxygen uptake data were recorded continuously from the warm-up until exhaustion was reached at 1-sec intervals using the Metabolism software (Pan Lab/Harvad Instruments, Spain). Exhaustion was assumed when the mice were not able to keep pace with the set treadmill speed (inability to stand still after being laid down). The VO_2_ max was achieved when the oxygen uptake plateaued despite increased treadmill speed.

After measuring the baseline VO_2_ max, the mice were randomly assigned to a sedentary control group (Control), which was limited to typical movement inside the cages, and the endurance training group (Trained). The endurance training group performed a four-week training protocol of running on the treadmill. During the four weeks, the mice ran five days per week for 1 h each day. During the first two weeks of training, the intensity was set at 70% VO_2_ max, and in the last two weeks, the intensity was set at 80% VO_2_ max.

All of the experiments described below were performed 24 h after the last training session, which even after considering the wide variation in exercise duration, intensity and type [13, 14], we considered enough to minimize the effects of the last session.

### Citrate synthase activity

After sacrifice, samples of the skeletal muscle were quickly removed and homogenised in an extraction solution buffer containing Tris–HCl (0.5 mM) and EDTA (1 mM) at a pH of 7.4. The reaction was performed in a medium containing Tris/aminomethane (100 mM), DTNB (0.2 mM), acetyl-CoA (0.1 mM), and Triton X-100 (0.1%) at a pH of 8.1. The reaction was initiated by the addition of 10 μL of the tissue extract and 50 μL of oxaloacetic acid (10 mM). Absorbance at 412 nm (25°C) was spectrophotometrically measured during 5 min as previously described [15].

### Tissue samples

Liver and muscle samples from Swiss mice were extracted at 15 min after an intraperitoneal injection of 1 IU/kg of total body weight of insulin and then were snap-frozen in liquid nitrogen and stored in -80for subsequent protein and mRNA extractions. Pancreatic islets were isolated from mice 24 h after the end of the training period by the collagenase method as previously described [16].

### Western blot

Western blots were performed as previously described [17].

### Pancreatic islet GSIS

Batches of 10 islets were pre-incubated for 1 h in *Krebs*-Henseleit buffer solution (KHBS) containing 0.5 g/l BSA and 5.6 mmol/l glucose and equilibrated at 95% O_2_ and 5% CO_2_ at 37°C. The medium was discarded, and the islets were incubated for an additional hour in 1 ml KHBS containing 2.8 or 16.7 mmol/l glucose. Subsequently, the supernatant fraction was collected to evaluate insulin secretion, and the remaining islets were homogenized in an alcohol/acid solution to measure the total insulin content by radioimmunoassay.

### Intraperitoneal glucose tolerance test

Swiss mice received an intraperitoneal injection of glucose (1 g/kg in 0.9% NaCl) after fasting for 8 h during dark cycle. Blood samples (25–50 μl) were collected from the tail immediately before the injection and 15, 30, 45, 60, 90 and 120 min afterwards to determine the glucose and insulin concentrations. The glucose concentration was measured using a glucose strip on an Accu-Chek Performa II instrument (Roche, Indianapolis, Indiana, USA), and the insulin concentration was determined by RIA as previously described [18].

### Intraperitoneal insulin tolerance test

Non-fasted Swiss mice received an intraperitoneal injection of insulin (1 U/kg). The blood glucose was measured using test strips (Accu-Chek Performa II) at baseline (0 min, before receiving insulin) and 5, 10, 15, 20, 30, 60 and 120 min after the administration of insulin. Glucose measurements were converted to natural logarithmic (Ln) values. The slope was calculated using linear regression (time × Ln[glucose]) and multiplied by 100 to obtain the glucose decay rate constant during the insulin tolerance test (*k*_ITT_, %/min).

### Insulin Decay

We determined the plasma insulin concentration in the Swiss mice that underwent an insulin tolerance test. The insulin decay was measured as previously described [19] using blood samples from 0, 5, 15 30 and 60 min after insulin administration. The rate constant for insulin loss (insulin decay) was calculated by converting the insulin measurements to natural logarithmic (Ln) values and calculating the slope using linear regression (time × Ln[insulin]); the results were multiplied by 100 to obtain the insulin decay rate constant (%/min).

### C-Peptide concentration and Insulin/C-Peptide ratio quantification

We determined the plasma C-Peptide concentration in the Swiss mice that underwent a glucose tolerance test. Blood samples were collected before glucose administration (0’) as well as 15’, 30’ and 60’ after. C-Peptide and insulin were measured from the same plasma sample. C-Peptide was evaluated using Rat/Mouse C-Peptide 2 ELISA Kit from Milipore (Cat. # EZRMCP2-21K) and insulin was assessed by RIA.

### Statistical analyses

Point-to-point comparisons were performed using Student’s *t*-test. For diferrent time points on the same animal, we applied the repeated measures anova. The groups were compared by t-test using GraphPad Origin 9.0 software. The results were considered significant if *p*<0.05.

## Results

### Metabolic variables

We double-checked the endurance training efficiency. First, by assessing physical and metabolic variables, we discovered that endurance training reduced total body weight, weight gain, perigonadal fat, and retroperitoneal fat and increased the time to exhaustion, speed, citrate synthase activity and VO_2_ max. Second, by evaluating glucose homeostasis-related parameters, we observed that the fasting and non-fasting plasma insulin and glucose levels were lower in the trained mice (Table 1).

**Table 1:** Metabolic variables of mice after endurance training.

VARIABLE	CONTROL	TRAINED
**Fasted Insulin (pmol/l)**	187.3±2.2	156.7±7.2*
**Non-Fasted Insulin (pmol/l)**	493.1±10.2	381.3±8.3*
**Fasted Glucose (mmol/l)**	7.76±0.50	6.21±0.41*
**Non-Fasted Glucose (mmol/l)**	10.1±0.21	8.41±0.45*
**Fasted C-Peptide (pmol/l)**	266.5±26.3	150.4±36.2*
**Non-Fasted C-Peptide (pmol/l)**	389.8±30.5	203.7±46.2*
**Fasted Insulin/C-peptide ratio**	0,78±0.18	1.11±0.51*
**Non-Fasted Insulin/C-peptide ratio**	1.04±0.14	1.79±0.24*
**Body Weight (g)**	39.8±1.02	36.4±0.51*
**Weight Gain (g)**	12±1.22	4.8±1.07*
**Perigonadal Fat (% of Total Weight)**	2.61±0.15	1.37±0.12*
**Retroperitoneal Fat (% of Total Weight)**	0.79±0.07	0.34±0.06*
**Citrate Synthase Activity (μmol.min** ^**-1**^ **.μg of Protein** ^**-1**^ **)**	0.28±0.06	0.41±0.06*
**Training Effectiveness—Resistance (seconds)**	225±14.2	390±25.1*
**Training Effectiveness—Speed (km/h)**	1.5±0.09	2.64±0.15*
**VO**_**2**_ **max Pre-Training (ml.min** ^**-1**^ **.kg** ^**-1**^ **)**	27.55±0.52	28.47±0.87
**VO**_**2**_ **max Post-Training (ml.min** ^**-1**^ **.kg** ^**-1**^ **)**	26.65±0.41	35.23±0.47*

The metabolic characteristics are presented for male Swiss mice after 4 weeks of endurance training. The values are reported as the mean ± SEM and were compared using a t-test (n = 4-6/group).

*significantly different from control mice

### Glucose tolerance and plasma insulin dynamics in Swiss mice

**Fig 1:**
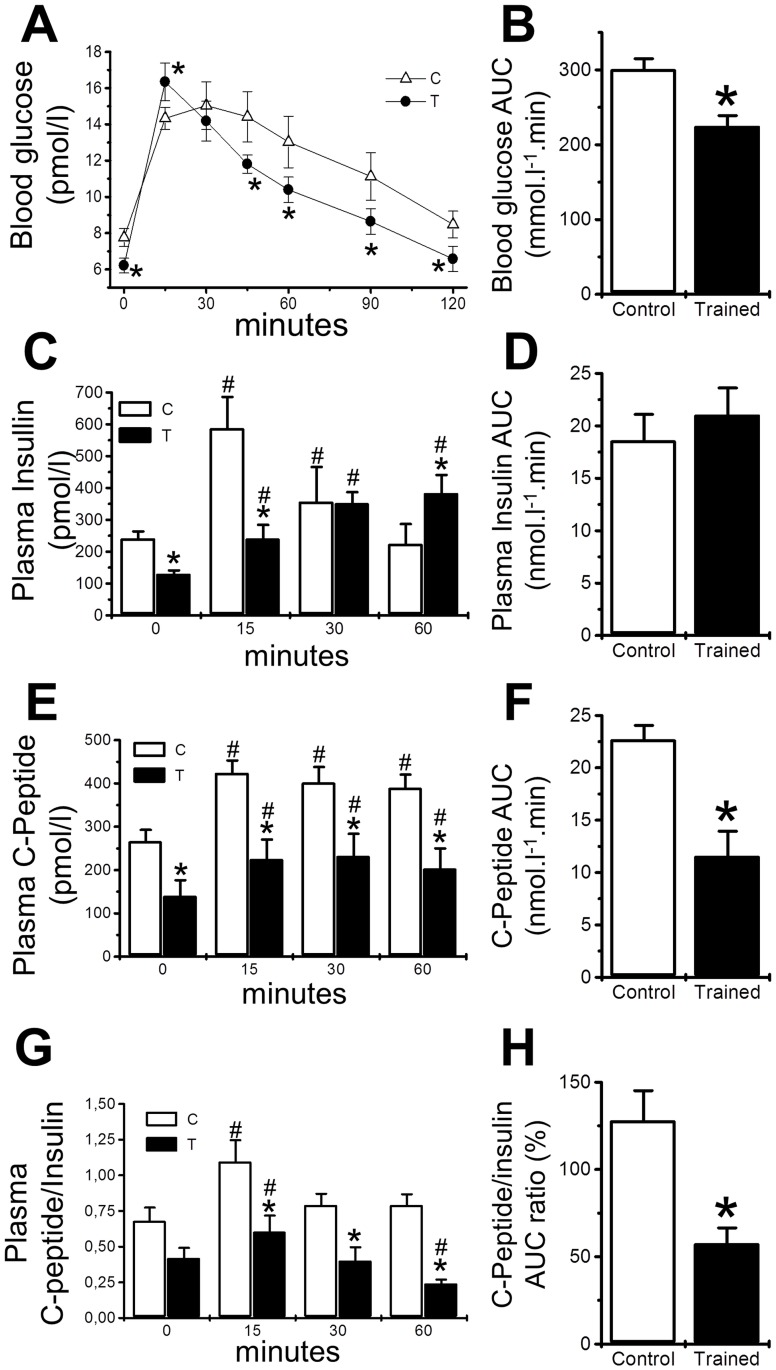
Endurance training effects over glucose tolerance and insulin dynamics of mice. The blood glucose (**A**) and plasma insulin (**C**) concentrations in 8 h-fasted, 28-day-old male Swiss mice 0, 15, 30, 45 and 60 min after the intraperitoneal injection of 1 g/kg glucose are presented for both the control (white triangle) and the trained (black circle) groups. The area under the curve (AUC) for blood glucose (**B**) and plasma insulin (**D**) is reported for 8 h-fasted, 28-day-old male Swiss mice after an intraperitoneal injection of 1 g/kg glucose. The plasma concentration of C-Peptide (**E**) and C-Peptide AUC (**F**) as well as C-Peptide/insulin ratio (**G**) and C-Peptide/insulin ratio AUC (**H**) in 8 h-fasted, 28-day-old male Swiss mice 0, 15, 30, and 60 min after the intraperitoneal injection of 1 g/kg glucose are presented for both the control (white triangle) and the trained (black circle) groups. The values are presented as the mean ± SEM and were compared using a repeated measures anova (n = 4-6/group). *significantly different from control mice (*p*<0.05)

Endurance training improved glucose tolerance (Fig. 1A and Fig. 1B) and reduced insulin concentration during the glucose tolerance test (Fig. 1C), but there was not a concomitant change in the overall (60 min) insulin concentration during this test (Fig. 1D). Therefore, endurance training altered insulin dynamics such that there was a lower insulin secretion peak at 15 min and a higher insulin concentration thereafter (Fig. 1C). C-Peptide (Fig. 1E) plasma concentrations were lower in trained mice at 15 min, but insulin concentration was higher in trained mice at 60 min and similar in 30 min, while the C-Peptide concentration remained lower in trained mice throughout the entire experiment (Fig. 1F), resulting in an overall reduced C-Peptide/insulin ratio in trained mice (Fig. 1G and Fig. 1H).

### Insulin secretion and total insulin content

Our results demonstrated that endurance training improved glucose tolerance and insulin sensitivity in mice. A potential mechanism for this effect is a training-induced improvement in insulin signaling in the liver and the skeletal muscle, but this does not explain the observed changes in the plasma insulin concentration and the insulin dynamics.

**Fig 2:**
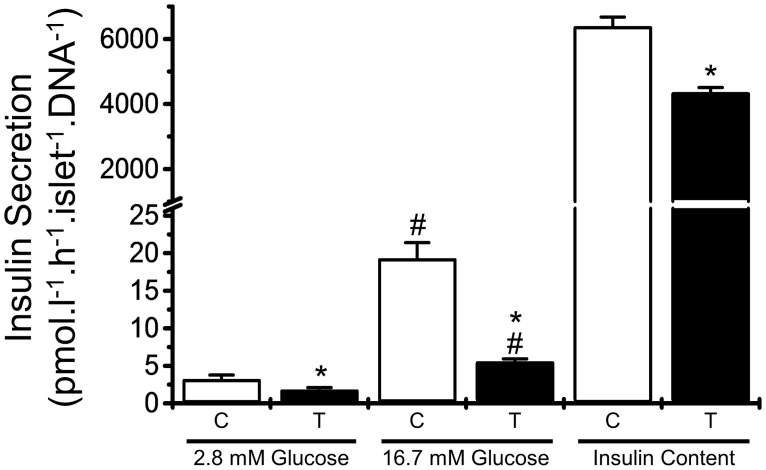
Endurance training effects over insulin secretion and content of pancreatic islets exposed to increasing glucose concentration. The ex-vivo insulin secretion and the total insulin content were measured in pancreatic islets that were isolated from control (white bars) and trained (black bars) 8-hour-fasted, 28-day-old Swiss mice and then incubated for 1 h in the presence of 2.8 or 16.7 mmol/l glucose. The values are presented as the mean ± SEM and were compared using a t-test (n = 8-12/group). *significantly different from control mice, #significantly different from the 2.8 mmol/l glucose condition (*p*<0.05)

To determine the origin of these physiological alterations, we analyzed the GSIS in islets isolated from both control and trained mice. Endurance training reduced insulin secretion at sub-stimulatory (2.8 mmol/l) and super-stimulatory (16.7 mmol/l) glucose concentrations and lowered the amount of insulin in the pancreatic islets (Fig. 2).

### Insulin sensitivity and decay in Swiss mice

**Fig 3:**
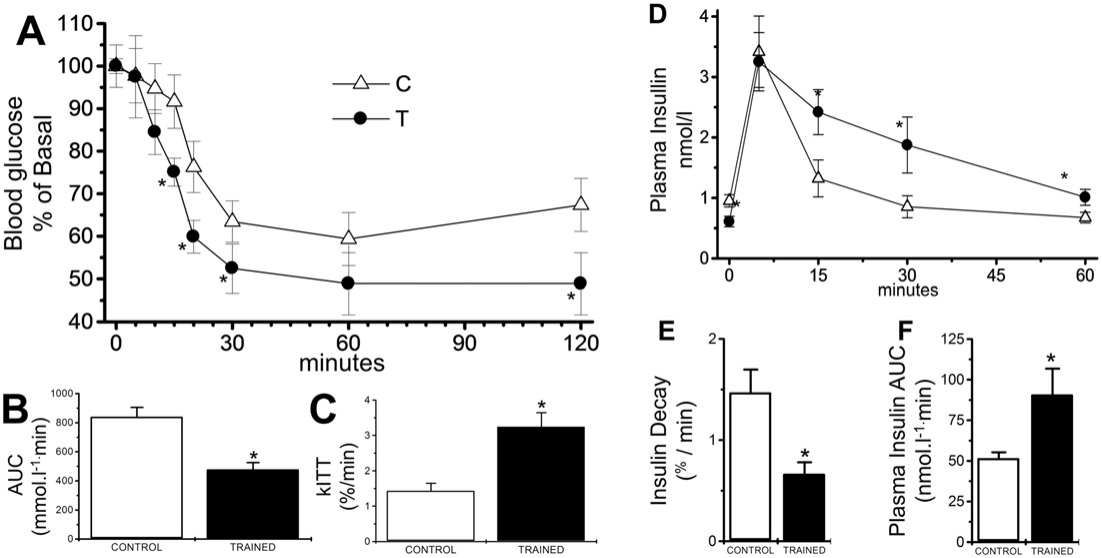
Endurance training effects over insulin sensitivity and decay of mice. The ITT results and the blood glucose concentrations at 0, 5, 10, 15 and 30 min (**A)**, the AUC for blood glucose (**B**), and the *k*_ITT_ (**C**) as well as the plasma insulin concentration at 0, 15, 30 and 60 min (**D)**, the AUC of insulin for the entire 60 min experiment (**E**) and the insulin decay over 60 min (**F**) after an intraperitoneal injection of 1 IU/kg insulin in non-fasted, 28-day-old male Swiss mice are presented.

Training-induced changes in glucose tolerance and insulin dynamics could be related to various factors, including insulin secretion, peripheral insulin sensitivity and insulin degradation. Insulin sensitivity was increased in trained Swiss mice as demonstrated by the lower insulin tolerance test (Fig. 3A and Fig. 3B) and the higher *k*_ITT_ (Fig. 3C). Endurance training reduced insulin degradation (Fig. 3D) and the insulin decay rate (Fig. 3E), resulting in an increased AUC of insulin during the 60 min experiment (Fig. 3F), suggesting a reduced insulin clearance in trained mice.

### Canonical (pIR and pAKT) and non-canonical (pCaMKII-pAMPK) pathway activation in the liver and the skeletal muscle of Swiss mice

**Fig 4:**
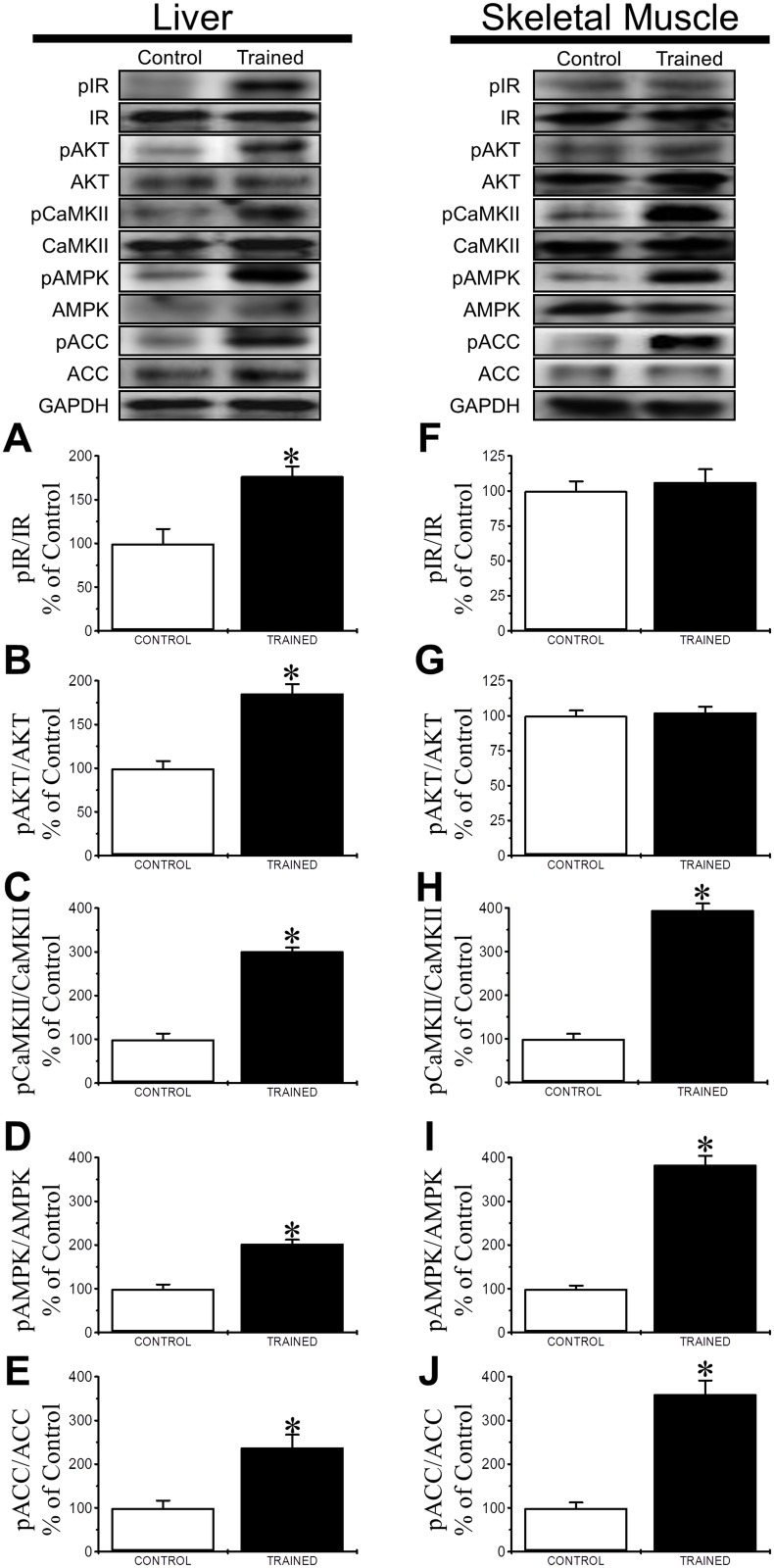
Endurance training effects over Canonical and Non-canonical insulin pathways in liver and skeletal muscle of mice. Illustrated are the phosphorylation of IR (**A**), AKT (**B**), CaMKII (**C**), AMPK (**D**) and ACC (**E**) in liver protein extracts and the phosphorylation of IR (**F**), AKT (**G**), CaMKII (**H**), AMPK (**I**) and ACC (**J**) in skeletal muscle protein extracts from both control (white bars) and trained (black bars) non-fasted 28-day-old male Swiss mice after an intraperitoneal dose of 1 IU/kg insulin. The values are presented as the mean ± SEM and were compared using a t-test (n = 4-6/group). *significantly different from control mice (*p*<0.05).

To evaluate the effects of endurance training on the canonical and non-canonical insulin pathways, we measured the phosphorylation of IR, AKT, CaMKII, AMPK and ACC in the liver and the skeletal muscle of Swiss mice. Before organ extraction, mice received 1 IU/kg of insulin. In the liver, training increased the phosphorylation of IR (Fig. 4A), AKT (Fig. 4B), CaMKII (Fig. 4C), AMPK (Fig. 4D) and ACC (Fig. 4E). In the skeletal muscle, endurance training did not affect the phosphorylation of IR (Fig. 4F) or AKT (Fig. 4G) but did increase the phosphorylation of CaMKII (Fig. 4H), AMPK (Fig. 4I) and ACC (Fig. 4J).

### Liver and skeletal muscle IDE expression

**Fig 5:**
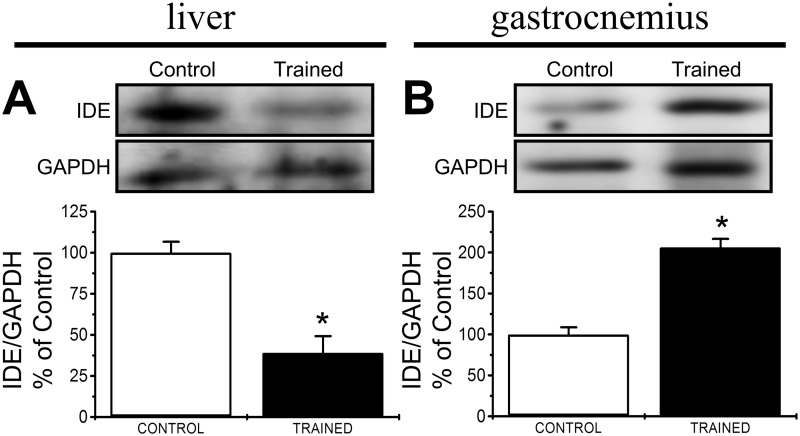
Endurance training effects IDE expression in liver and skeletal muscle. The expression of IDE in protein extracts from the liver (**A**) and the skeletal muscle (**B**) of control (white bars) and trained (black bars) non-fasted, 28-day-old male Swiss mice is depicted. The values are presented as the mean ± SEM and were compared using a t-test (n = 8-12/group) *significantly different from control mice (*p*<0.05)

We determined how endurance training reduced insulin degradation in vivo by evaluating IDE expression in the liver and the skeletal muscle. Endurance training reduced the protein expression of IDE in the liver (Fig. 5A) and increased the amount of IDE protein in the skeletal muscle (Fig. 5B).

## Discussion

It is well established that three major processes control blood glucose through insulin: insulin secretion (the ability of pancreatic islets to secrete adequate amount of insulin), insulin sensitivity (the response of peripheral organs to insulin) and insulin clearance (the rate at which insulin is removed from the plasma)[20].

During and immediately after exercise, the increase in glucose uptake by the skeletal muscle in healthy subjects is IR–AKT pathway independent [21] and involves AMPK activation [22], the same process occurs in trained subjects, even in the rest period after the last session of exercise [14]. During muscular contraction, the concentration of ATP in muscle is kept constant whereas the AMP concentration increases and it’s related with the duration and intensity of exercise, increasing AMPK activity [23, 24]. The mechanism by which AMP may regulate AMPK activity is not completely known. Upstream proteins as LKB1 and CAMKII might have a crucial role in this context.

Here, we reported higher expression and phosphorylation of CAMKII and AMPK proteins showing that our training protocol was effective to induce these molecular adaptations [14, 24, 25]. Despite the possible skeletal muscle and pancreas crosstalk [26, 27], the improved insulin sensitivity does not directly justify the lower plasma insulin levels in the trained mice, so we evaluated the insulin secretion from pancreatic islets and the hepatic insulin clearance, whose combined effects could account for the plasma insulin dynamics observed.

Endurance training reduced GSIS in the isolated pancreatic islets, which reduced the degree of insulinemia that was observed in the trained mice, but this effect is unlikely to completely account for the observed physiological differences. The islets from trained mice secreted ~50% less insulin than did the control islets in response to sub- and supra-stimulatory glucose concentrations (Fig. 2). However, the plasma insulin concentration in fasted and non-fasted mice was only ~25% lower (Table 1). After secretion, insulin is removed from the plasma by the liver [20] in a process termed insulin clearance, which is altered in obesity [28] and models of type 2 diabetes [2, 29, 30]; the dysregulation of insulin clearance is commonly associated with obesity [31, 32] and type 2 diabetes in humans [2, 33, 34].

It is the standard procedure in clinical practice to consider the plasma insulin levels as the rough equivalent to insulin secreted by pancreatic islets β cells. Although this might be the case in many instances, the two variables are hardly substitutes, because plasma insulin level is the end result from the balance between pancreatic islets insulin secretion and liver insulin clearance. Insulin is secreted together with a byproduct of its synthesis and processing, the C-Peptide, in a 1:1 ratio, but despite having the same rate of secretion, C-Peptide in removed from plasma at a severely lower rate than insulin, so that any change in the ratio of C-Peptide to insulin in plasma is mainly subjacent to alterations in the rate of insulin removal, or insulin clearance. Although it is not the gold standard methodology, the C-Peptide/Insulin plasma ratio during GTT is a reliable widespread and accepted overall as an indicator of insulin clearance [35–37].

The importance of insulin clearance in the exercise-induced modulation of glucose is unclear. In type 2 diabetes, insulin clearance is impaired and is associated with insulin resistance and the loss of beta cell mass. However, it is not known whether this reduction is causative and precedes the other symptoms [2, 29, 38] or is a compensatory mechanism that is activated to ameliorate the effects of the reduced insulin secretion and the increased insulin resistance [19, 39, 40].

Endurance training reduced insulin clearance, decay and AUC and increased peripheral insulin sensitivity (Fig. 3), which were confirmed by the reduced C-Peptide/insulin ratio during GTT (Fig. 1). This indicates that reduced insulin clearance is a compensatory mechanism that allows the organism to overcome insulin resistance, which attenuates the beta cell overload and increases their survival, thus delaying and/or ameliorating type 2 diabetes.

Intracellular insulin degradation depends on IDE activity [20, 41]. IDE is a 110 kDa zinc-dependent metalloproteinase that is expressed in most insulin-responsive cells and is most highly expressed in hepatocytes. Given the importance of insulin clearance in type 2 diabetes and the role of IDE in its regulation, it is not surprising that alterations in the expression and the activity of IDE are closely related to the onset and development of type 2 diabetes [34, 41]. Therefore, we evaluated whether endurance training controlled insulin degradation by measuring *IDE* expression in the liver and the skeletal muscle. In trained mice, the expression of *IDE* was decreased in the liver (Fig. 5A) and increased in the skeletal muscle (Fig. 5B). The central role of hepatocytes in insulin degradation [20, 42, 43] and the reduced expression of IDE in the liver contribute to the decreased insulin clearance in the trained mice. Although IDE expression can be considered the main factor for its activity, some factors are known to affect its activity, such as ATP levels [44] or even Redox agents [45, 46], which does not detract from our observations about IDE expression importance to the process of insulin clearance, but are important to note regardless.

The mechanism by which IDE expression is increased in the skeletal muscle is more complex. We hypothesize that insulin degradation is elevated in skeletal muscle to maintain adequate glucose uptake in an environment of increased insulin sensitivity; in this way, glucose levels are maintained independently of other organs, where insulin remains essential for glucose uptake.

In contrast to skeletal muscle, the canonical pathway that is responsible for the boost in glucose uptake was increased in the liver. We propose that the difference in the insulin response between the liver and the skeletal muscle of trained mice is due to the opposite effects of training on the expression of IDE in these two organs. Inhibiting IDE improves insulin sensitivity through increased phosphorylation of IR [47]. CNTF, an anti-obesity, anti-diabetogenic cytokine [16, 18, 48–50], promotes the same effects in mice: reduced IDE expression in the liver, reduced insulin clearance, and improved insulin sensitivity through IR-AKT phosphorylation.

Therefore, the increased expression of IDE in the skeletal muscle may compensate for any increase in IR-AKT phosphorylation that is induced by the training exercise, thus resulting in the unaltered state that we observed.

In the mice, exercise reduced insulin secretion and degradation, which helped maintain normoglycemia without taxing the pancreatic beta cells. Inhibiting IDE expression in hepatocytes increases IR phosphorylation and insulin sensitivity [47].

The critical role of exercise in the prevention and the management of type 2 diabetes is undisputed. Lifestyle interventions, including diet and physical activity, reduce the incidence of diabetes in 60% of subjects with impaired glucose tolerance [51]. In addition to its effect on insulin sensitivity, exercise improves hemoglobin A1C, maximal oxygen consumption (VO_2_ max) and muscle microvascular perfusion and reduces body fat [12]. The latter is particularly important because it has been estimated that 80% of the newly diagnosed cases of type 2 diabetes are due to obesity [52, 53].

In summary, our results demonstrated that endurance training reduced insulin clearance in mice, probably by downregulating the expression of IDE in the liver, effects that were accompanied by increased insulin sensitivity, thereby revealing another beneficial effect of physical exercise on glycemic control.
